# Creutzfeldt-Jakob Disease: Alterations of Gut Microbiota

**DOI:** 10.3389/fneur.2022.832599

**Published:** 2022-04-15

**Authors:** Yanjun Guo, Yichen Xu, Xue Lin, Zhen Zhen, Fang Yi, Hongzhi Guan, Qi Shi, Wenjie Sun, Anchao Yang, Xiaoping Dong, Jiawei Wang

**Affiliations:** ^1^Department of Neurology, Beijing Tongren Hospital, Capital Medical University, Beijing, China; ^2^Department of Neurosurgery, Beijing Tiantan Hospital, Capital Medical University, Beijing, China; ^3^Department of Neurology, Lishilu Outpatient, Central Medical Branch of PLA General Hospital, Beijing, China; ^4^Department of Neurology, Peking Union Medical College Hospital, Chinese Academy of Medical Sciences, Beijing, China; ^5^State Key Laboratory for Infectious Disease Prevention and Control, National Institute for Viral Disease Control and Prevention, Chinese Center for Disease Control and Prevention, Beijing, China; ^6^Department of Hematology, Peking Union Medical College Hospital, Chinese Academy of Medical Sciences, Beijing, China

**Keywords:** Creutzfeldt-Jakob disease, gut microbiota, dysbiosis, neurodegenerative disease, gut-brain axis

## Abstract

**Introduction:**

Human gut dysbiosis has been implicated with the onset of many neurodegenerative disorders. However, the current data focused on the gut microbiota of patients with Creutzfeldt-Jakob disease (CJD) are still lacking. In our study, we explored the gut microbiota alteration in patients with CJD.

**Method:**

We performed 16S ribosomal RNA MiSeq sequencing in stool samples of patients with CJD and controls. Functional analysis of the gut microbiota between these two groups was based on Kyoto Encyclopedia of Genes and Genomes and Phylogenetic Investigation of Communities by Reconstruction of Unobserved States 2. Clinical rating scales were used to evaluate the association between cognitive impairment and gut microbiota alteration.

**Result:**

We identified a significant alteration in both the structure and the richness of the CJD group. Function analysis revealed that the gut microbiota of patients with CJD enriched in immune signaling molecule interactions and xenobiotics biodegradation. MoCA and survival times were found to be associated with gut microbiota in patients with CJD.

**Conclusion:**

We demonstrated an altered gut microbiota in patients with CJD, which was associated with the cognitive impairment and the survival time of these patients.

## Introduction

Creutzfeldt-Jakob disease (CJD), a common form of human prion disease, is a rare neurological disorder with an incidence of 1–2 cases per million per year (1). CJD can be classified as sporadic CJD (sCJD), genetic CJD (gCJD), or acquired CJD. sCJD is the most common type of CJD, accounting for 85% of all cases (2). CJD originates from the aggregation of the abnormal form of prion protein (PrP^Sc^) in the brain, which causes cerebral spongiform formation, neural loss, and astrogliosis (3). CJD often manifests as rapidly progressive dementia of unknown origin, accompanied by myoclonus, visual changes, ataxia, and akinetic mutism (4). Despite the unprecedented efforts in CJD research, the mean survival time of patients with CJD remains only at 4–8 months (5, 6). Alzheimer's disease (AD) and Parkinson's disease (PD) are common forms of neurodegenerative disorders, and they share similar presentations as CJD. The critical pathological events in AD and PD include the accumulation of amyloid-beta (Aß) and alpha-synuclein (α-syn), respectively, which have been found to possess prion-like characteristics (7–10). All these studies indicate homology between CJD and AD/PD, and we believe the research in AD and PD may guide the study of CJD.

In recent years, the gut microbiota has become a research hot spot in the field of neurodegenerative disorders since the gut is deeply interconnected with the brain (11, 12). The gut contains one of the most complex reservoirs of microbes, the dysbiosis of which is involved in multiple human diseases (13–16). Significant gut microbe composition alterations have been reported in both AD and PD (17–19). For example, there is a reduced proportion in phylum Firmicutes and Actinobacteria, and an enriched proportion in phylum Bacteroidetes and Proteobacteria, in patients with AD (20, 21). In addition, in patients with PD, genus Ralstonia is markedly increased, while a decrease is observed in genus Blautia, Coprococcus, and Roseburia when compared to healthy control (18). Since CJD and AD/PD are all neurodegenerative disorders characterized by the accumulation of misfolded protein, we believe that gut microbiota dysbiosis may play a role in the development of CJD. Recently, Yang et al. reported significant alterations in both the structure and abundance of the gut microbiota in the mice with prion disease (22). However, data focused on the gut microbiota of humans with prion disease have not been reported yet.

In this study, we recruited 10 patients with CJD and 10 matched healthy controls to perform 16S ribosomal RNA (rRNA) gene sequencing of DNA isolated from fecal samples to characterize the microbe structures. We also analyzed the correlation of the gut microbe with CJD clinical profiles, such as survival time, central spinal fluid (CSF) 14-3-3 protein, and rating scales, namely, Mini-Mental State Examination (MMSE), Montreal Cognitive Assessment (MoCA), and Clinical Dementia Rating scale Sum of Boxes (CDR-SB). We hope that unveiling the difference between patients with CJD and healthy individuals in regards to the gut microbiota may provide new insights into the management and treatment of patients with CJD. To our knowledge, there are currently no studies directly addressing the alteration of gut microbiota composition among patients with CJD and healthy controls.

## Method

### Patient Description

All subjects who participated in this study were well-informed about the purpose of the study, and all the subjects provided written consent forms. The study was approved by the ethics committee of Beijing Tongren Hospital, Capital Medical University, Beijing, China.

We recruited 10 patients with CJD and 10 gender and age-matched controls. The patients were diagnosed with CJD before being recruited to the study. Three patients with CJD (ZJ, CYH, and SXQ) had a mutation in the PRNP gene (ZJ and CYH had G114V mutation; SXQ had T118K mutation) and were diagnosed as gCJD (23). The other patients were diagnosed as sCJD based on accepted standard criteria released in 2017 (6). One of our patients with CJD was diagnosed at an advanced stage (CDH), while the rest of our patients were diagnosed at an early stage. Patients diagnosed as definite gCJD and probable sCJD were included in this study. The exclusion criteria were as follows: (1) antibiotics or probiotics were used within 2 weeks before the study; (2) in severe malnutrition or infection condition; (3) addicted to drugs, alcohol, or smoking; (4) had a history of bowel syndrome that include constipation, diarrhea, and/or other bowel diseases 1 year before the study; and (5) have diabetes, hypertension, or other multisystem diseases.

Before we collected fecal samples, the participants were asked to complete a questionnaire including diet changes, smoking, alcohol intake, etc., for 7 days before fecal sample collection. None of the patients reported changes in diet or medication.

### Stool Sample Collecting and Bacterial 16S rRNA Amplification

The stool sample was collected using E.Z.N.A.^®^ Stool DNA Kit when patients were admitted to the outpatients for routine reexamination. The sample was then kept at −20°C in a portable refrigerator. The samples were then transported to the laboratory within 24 h at 4°C. In the laboratory, the stool samples were stored at −20°C in a refrigerator before further analysis. The doctors who collected the stool samples and the researchers who analyzed the stool sample in the laboratory were blind to the study design and the diagnosis of the patients.

Stool DNA was extracted using PowerSoil DNA Isolation Kit (MoBio Laboratories, Carlsbad, CA, USA) following the manual. In brief, a 200 mg stool sample was used for DNA extraction. The stool sample was mixed with SLX-Mlus Buffer for homogenization. The sample was then centrifuged with the addition of 180 μl DS buffer and the supernatant was collected. cHTR reagent and P2 buffer were added to remove the inhibitory substances from stool samples. Furthermore, proteinase K was used to rapidly inactivate the nucleases which otherwise may degrade the DNA during purification. Finally, the DNA samples were collected in the HiBind® DNA Mini Column and eluted *via* elution buffer. The purity and quality of the genomic DNA were checked on 1% agarose gels and a NanoDrop spectrophotometer (Thermo Scientific). A total of 30 ng of the DNA sample was taken for further PCR amplification. The 16S V3-V4(336-806) hypervariable region of the bacterial 16S rRNA gene was amplified with the primers 5′-GTACTCCTACGGGAGGCAGCA-3′ and 5′-GTGGACTACHVGGGTWTCTAAT-3′. For each stool sample, an 8-digit barcode sequence was added to the 5′ end of the forward and reverse primers (provided by Allwegene Company, Beijing). The PCR was carried out on a Mastercycler Gradient (Eppendorf, Germany) using 25 μl reaction volumes, containing 12.5 μl 2 × Taq PCR MasterMix, 3 μl BSA (2 ng/μl), 1 μl forward primer (5 μM), 1 μl reverse primer (5 μM), 2 μl template DNA, and 5.5 μl ddH_2_O. Cycling parameters were 95°C for 5 min, followed by 28 cycles of 95°C for 45 s, 55°C for 50 s, and 72°C for 45 s with a final extension at 72°C for 10 min. The PCR products were purified using an Agencourt AMPure XP Kit. After we harvested the PCR products, deep sequencing was performed. The sequencing was done by the Miseq platform at Allwegene Company (Beijing). After the run, image analysis, base calling, and error estimation were performed using Illumina Analysis Pipeline Version 2.6.

### Data Analyses

The raw data were first screened, and the bad sequences were removed before being separated based on the sample-specific barcode. Data were considered bad if they were shorter than 230 bp, had a low-quality score (≤ 20), contained ambiguous bases, or did not exactly match primer sequences and barcode tags. Finally, the qualified reads with 97% similarity were clustered into operational taxonomic units (OTUs) using the Uparse algorithm of Vsearch (v2.7.1) software (24). To identify the richness and diversity of the sample, abundance-based coverage estimator, Chao1, and inverse Simpson and Shannon index were calculated based on the OTU information in QIIME (v1.8.0). To compare the richness and diversity difference between the patient group and control group, a Turkey test was performed and a significant difference was considered when *p* value <0.05. Furthermore, the Ribosomal Database Project (RDP) Classifier tool was used to classify all sequences into different taxonomic groups based on the SILVA128 database (25). Based on the results of taxonomic annotation and relative abundance, R (v3.6.0) software was used for bar-plot diagram analysis. We also used the mixOmics package in R (v3.6.0) software to perform the partial least squares discrimination analysis (PLS-DA) to examine the microbiota community structure between different samples, based on the OTU information from each sample (24). Wilcoxon test was performed using stats package in R (v3.6.0) to determine the microbiota with different abundance in various taxa between patient group and control group. The linear discriminant analysis with effect size (LEfSe) characterized the taxa with statistical significance and biological relevance. Python (V2.7) was used to perform the LEfSe analysis and we set LDA score > 3 as a threshold.

To better understand the altered gastrointestinal microbiota community, a functional predictive analysis was performed based on Kyoto Encyclopedia of Genes and Genomes (KEGG) using Phylogenetic Investigation of Communities by Reconstruction of Unobserved States 2 (PICRUSt2, v2.3.0). We also explored the relationship between CSF 14-3-3 protein, rating scales, namely, MMSE, MoCA, and CDR-SB with the gastrointestinal microbiota. MMSE was used to assess the cognitive status of the patient. We used a Beijing version of MoCA in our study, as it is most wildly accepted in China. CDR-SB was used to evaluate the degree of dementia. CSF fluid was taken in the morning after a 12-h fast *via* a lumbar puncture at the L4 and L5 interspaces. This sample was sent to the laboratory immediately for 14-3-3 protein detection. All patients completed the rating scale the first time they were admitted to the hospital, and it was performed by the same neurologist. The correlation between CSF 14-3-3 protein and rating scale was calculated by the Spearman algorithm, and the correlation heatmap was drawn by ggplot2 package in R (v3.6.0) software.

### Data Availability

Anonymized data not published within this article will be made available by request from any qualified investigator.

## Result

### Study Subject Description

The patients and healthy individuals as control were recruited from Beijing Tongren Hospital, Capital Medical University, and all were informed of the study design and provided written consent forms. The detailed information of the participants is shown in Table 1. We recruited 10 patients with CJD and 10 gender and age-matched controls (average age: CJD group vs. control group, 57.1 ± 11.5 vs. 58.2 ± 11.6, respectively, *p* value = 0.84). Of the recruited patients, dementia (90%, 9/10), rigidity (60%, 6/10), ataxia (50%, 5/10), and myoclonus (40%, 4/10) were the most frequent clinical manifestation in our patients with CJD, which is consistent with the literature (26).

**Table 1:** Summary of the participants.

	**CJD patients**	**Healthy controls**	***p*** **-value**
*n*	10	10	
Age (yrs, mean ± SD)	57.1 ± 11.5	58.2 ± 11.6	0.84
Sex (% male)	60% (6/10)	60% (6/10)	
Survival duration (month, mean ± SD)	9.7 ± 6.8		
MMSE (mean ± SD)	18.7 ± 7.4		
MoCA (mean ± SD)	12.3 ± 7.2		
CDR-SB (mean ± SD)	8.3 ± 5.7		
BMI (kg/m^2^, mean ± SD)	23.8 ± 1.6	23.4 ± 1.2	0.37
Clinical symptoms			
Dementia	90% (9/10)		
Hallucinations	20% (2/10)		
Myoclouns	40% (4/10)		
Rigidity	60% (6/10)		
Ataxia	50% (5/10)		
Dystonia	0% (0/10)		
Akinetic Mutism	10% (1/10)		

Gene analysis revealed that of the 10 patients, 3 had a mutation in the PRNP gene (2 patients had G114V mutation and 1 patient had T188K mutation). Subsequently, these patients were diagnosed with gCJD.

In addition to these disease-related mutations, single nucleotide polymorphisms at codons 129 and 219 of the PRNP gene represent susceptibility factors for human prion diseases. All 10 patients with CJD exhibited methionine/methionine homozygote at codon 129 (M129M). An overwhelming percentage of East Asians (92–94%) exhibit M129M, but at a much lower percentage compared with Caucasians that have this polymorphism (32–45%).

By the time of drafting this manuscript, 7 patients with CJD had died. The mean survival time of these patients was 9.7 ± 6.8 months. The remaining 3 patients are still alive and have survived for more than 1 year. Remarkably, 2 of these 3 patients have survived for more than 2 years, which is longer than the average survival time for most patients with CJD (6, 27).

### Alterations of Microbiota Structure in CJD Patients and Healthy Controls

**Figure 1:**
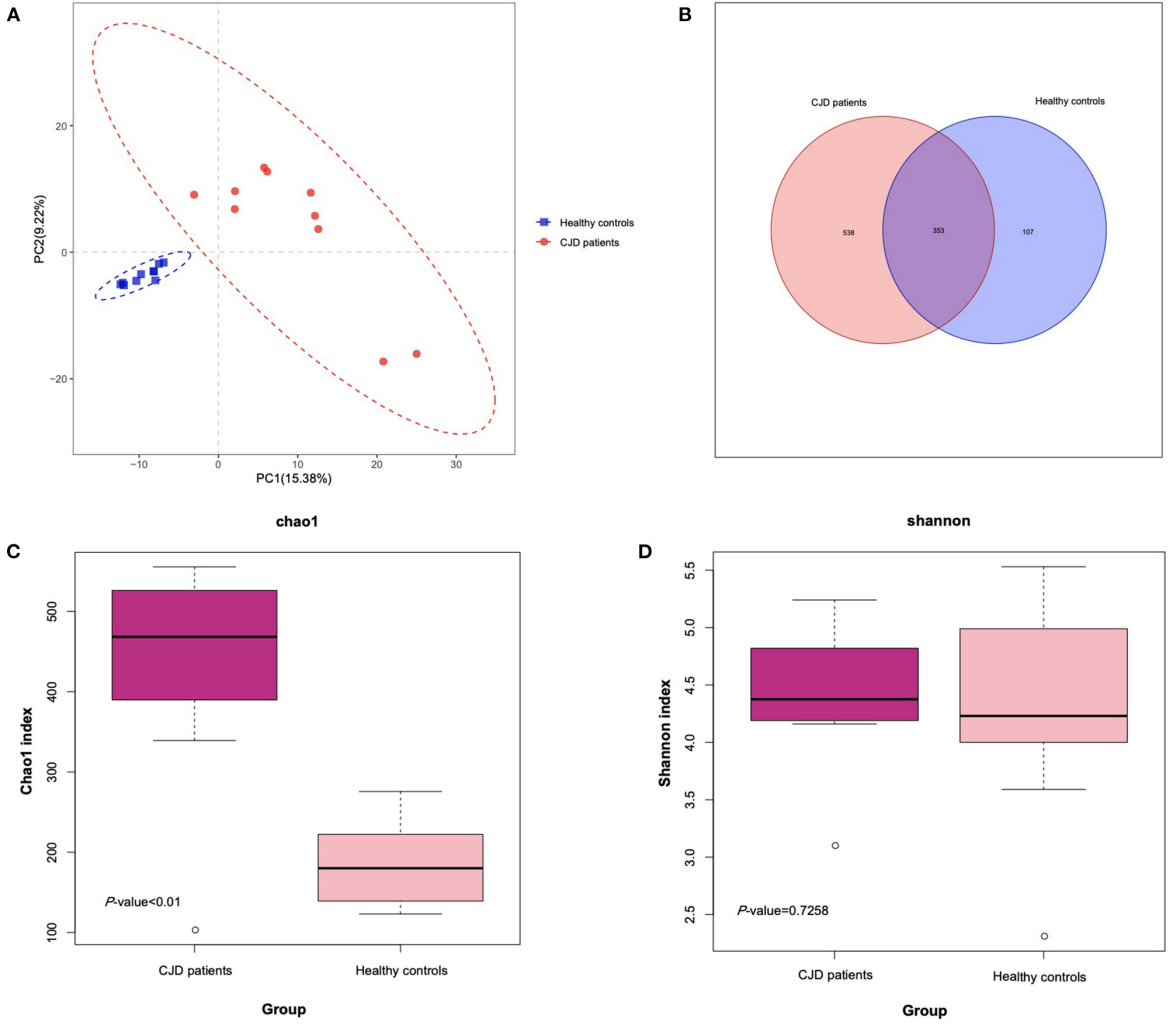
Comparison of the microbial diversity of patients with CJD and control. **(A)** PLS-DA analysis of the patients with CJD and control using R software. The CJD group and control group were separated without overlap, suggesting a distinct gut microbiota community in CJD and control groups. **(B)** Venn diagram illustrates the OTUs' overlap of fecal samples from CJD and control. **(C)** Box plot illustrates significant difference (*p* < 0.05) of Chao1 index in the 2 groups. Median, interquartile range, minimum, and maximum values are shown in the box plot. **(D)** Box plot illustrates nonsignificant difference (*p* = 0.73) of Shannon index in the 2 groups. Median, interquartile range, minimum, and maximum values are shown in the box plot.

To identify the structural alterations between patients with CJD and controls, we perform 16s rRNA sequencing. The sequencing reads were flattened and then clustered into OTUs at 97% similarity. As shown in the Venn diagram of Figure 1B, we finally identified a total of 998 OTUs in all samples, among which 353 OTUs were shared by both the CJD and control groups. The gut microbiota diversity was determined *via* α-diversity, namely, Chao1, Shannon's index, observed species, PD whole tree, and β-diversity, namely, PLS-DA. As shown in Figures 1C,D, the CJD group presented a significantly higher Chao1 index compared to the control group (CJD group vs. healthy controls: 433.2 vs. 187.0, *p* value < 0.01), indicating a higher microbiota community richness in the CJD group. Conversely, the Shannon index was similar in both groups (CJD group vs. healthy controls: 4.4 vs. 4.3, *p* value = 0.73). The CJD group also showed a significant increase in observed species and PD whole tree compared to the healthy controls (Supplementary Figure). Furthermore, the microbiota community was greatly separated in PLS-DA analysis (Figure 1A), indicating a unique microbiota community in the CJD group.

### Key CJD Associated Gut Microbiota

**Figure 2:**
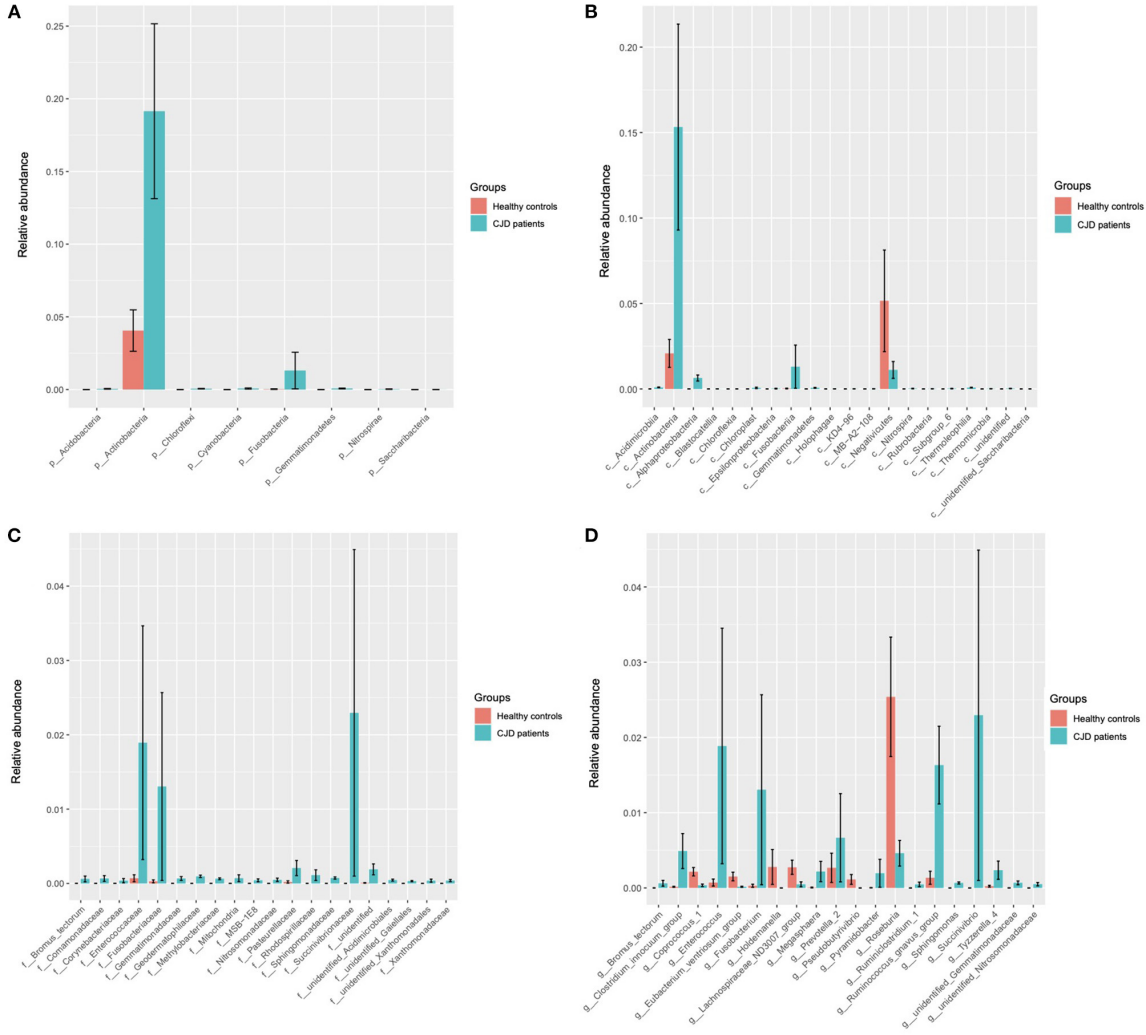
Wilcoxon test showed the key microbiota that was significantly different in the CJD group (sgroup) and control group (cgroup) at different biological taxonomy: phyla **(A)**, class **(B)**, family **(C)**, and genus **(D)**. **(A)** At the phyla level, Actinobacteria and Fusobacteria were significantly increased in the CJD group. **(B)** At the class level, we found a significant enrichment of Fusobacteriia, Actinobacteria, and Alphaproteobacteria in the CJD group. Conversely, we found a decrease of Negativicutes in the CJD group compared to the control group. **(C)** At the family level, significant increases were found in Fusobacteriaceae, Bifidobacterium, Succinivibrionaceae, and Enterococcaceae within the CJD group. **(D)** At the genus level, Fusobacterium, Succinivibrio, Enterococcus, *Ruminococcus gnavus* group, and Tyzzerella 4 were significantly increased in the CJD group, and Coprococcus 1, Lachnospiraceae_ND3007, Pseudobutyrivibrio, Roseburia, and Holdemanella were decreased in the CJD group. p, phylum; c, class; o, order; f, family; g, genus.

A Wilcoxon test analysis was performed to identify the key gut microbiota in the patient with the CJD group, and we found a significant alteration at a board taxonomic level (Figure 2 and Table 2).

**Table 2:** Gut microbiota with significant alteration in CJD patients.

	**Gut microbiota that increases in CJD patients**				**Gut microbiota that decreases in CJD patients**			
**Taxonomic**	**Microbiota**	**CJD**	**Control**	***p*** **-value**	**Microbiota**	**CJD**	**Control**	***p*** **-value**
Phylum	p__Actinobacteria	0.191446873	0.040547287	0.03				
	p__Fusobacteria	0.013046345	0.000282592	0.01				
Class	c__Actinobacteria	0.153235682	0.020827054	0.03	c__Negativicutes	0.011138847	0.051577807	0.02
	c__Fusobacteriia	0.013046345	0.000282592	0.01				
	c__Alphaproteobacteria	0.006424265	0.0000141	<0.01				
Family	f__Enterococcaceae	0.018938395	0.000706481	<0.01				
	f__Fusobacteriaceae	0.013046345	0.000282592	0.01				
	f__Succinivibrionaceae	0.022951206	0.000	<0.01				
	f__Bifidobacteriaceae	0.150122457	0.020732856	0.04				
Genus	g__Fusobacterium	0.013046345	0.000282592	0.01	g__Coprococcus_1	0.000343821	0.002147702	0.04
	g__Succinivibrio	0.022951206	0.000	0.02	g__Pseudobutyrivibrio	0.000	0.001125659	0.03
	g__Enterococcus	0.018863037	0.000706481	<0.01	g__Roseburia	0.004606255	0.025395629	0.04
	g__Ruminococcus _gnavus_group	0.016319706	0.001347023	<0.01	g__Holdemanella	0.0000	0.002783534	0.03
	g__Tyzzerella_4	0.002350226	0.000226074	0.03	g__Lachnospiraceae _ND3007_group	0.000470987	0.002741145	0.02
	g__Prevotella_2	0.006664469	0.002670497	0.03				

At the phylum level, 2 main phyla, namely, Actinobacteria (CJD group vs. healthy controls: 0.19 vs. 0.04, *p* value = 0.03) and Fusobacteria (CJD group vs. healthy controls: 0.01 vs. 0.00, *p* value = 0.01) increased significantly in the CJD group. However, we did not observe an increase in phylum Proteobacteria (CJD group vs. healthy controls: 0.07 vs. 0.07, *p* value = 0.22), although its class, family, and genus were increased markedly in the CJD group. We identified a higher Firmicutes: Bacteroides ratio in the CJD group, though it did not reach the level of significance (CJD group vs. healthy controls: 8.24 vs. 3.03, *p* value = 0.25).

At the class level, we identified significant increases in Fusobacteriia (CJD group vs. healthy controls: 0.01 vs. 0.00, *p* value = 0.01), Actinobacteria (CJD group vs. healthy controls: 0.15 vs. 0.02, *p* value = 0.03), Alphaproteobacteria (CJD group vs. healthy controls: 0.01 vs. 0.00, *p* value < 0.01). Conversely, we observed a decrease in Negativicutes (CJD group vs. healthy controls: 0.01 vs. 0.05, *p* value = 0.02) within the CJD group compared to healthy controls.

At the family level, there was a higher abundance in Fusobacteriaceae (CJD group vs. healthy controls: 0.01 vs. 0.00, *p* value = 0.01), Succinivibrionaceae (CJD group vs. healthy controls: 0.02 vs. 0.00, *p* value < 0.01), Enterococcaceae (CJD group vs. healthy controls: 0.02 vs. 0.00, *p* value < 0.01) in the CJD group. Furthermore, Bifidobacteriaceae also showed an increased in the CJD group (CJD group vs. healthy controls: 0.15 vs. 0.02, *p* value = 0.05).

At the genus level, an enrichment in the abundance of Fusobacterium (CJD group vs. healthy controls: 0.01 vs. 0.00, *p* value = 0.01), Succinivibrio (CJD group vs. healthy controls: 0.02 vs. 0.00, *p* value = 0.02), Enterococcus (CJD group vs. healthy controls: 0.019 vs. 0.000, *p* value < 0.01), Ruminococcus gnavus group (CJD group vs. healthy controls: 0.016 vs. 0.001, *p* value < 0.01), Tyzzerella 4 (CJD group vs. healthy controls: 0.002 vs. 0.000, *p* value = 0.03) was seen in the CJD group. While a decrease was observed in the abundance of Coprococcus1 (CJD group vs. healthy controls: 0.000 vs. 0.002, *p* value = 0.04), Lachnospiraceae_ND3007 (CJD group vs. healthy controls: 0.000 vs. 0.002, *p* value = 0.02), Pseudobutyrivibrio (CJD group vs. healthy controls: 0.000 vs. 0.001, *p* value = 0.03), Roseburia (CJD group vs. healthy controls: 0.004 vs. 0.025, *p* value = 0.04), and Holdemanella (CJD group vs. healthy controls: 0.000 vs. 0.003, *p* value = 0.03) in the CJD group compared to the controls.

**Figure 3:**
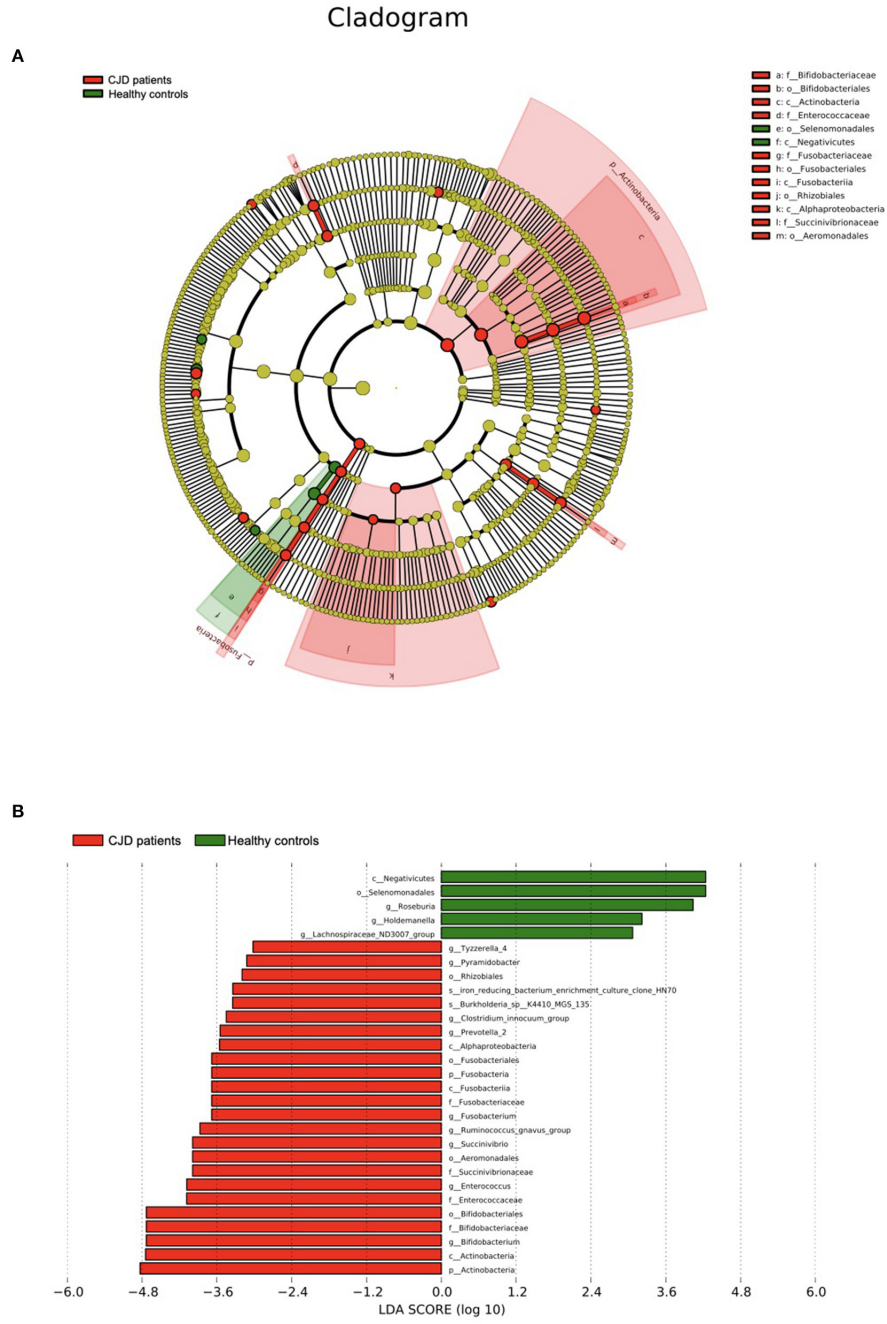
Results of the LEfSe using Python (V2.7). **(A)** The outer ring to the inner ring of the cladogram represents the biological taxonomy from phyla to genus. Each dot in the ring represents a microbiota in the corresponding taxonomy level, and the diameter of each dot is proportional to the taxon's abundance. The color of each dot represents the difference in the abundance in the corresponding microbiota between patients with CJD and controls. Yellow indicates the difference in the abundance between the 2 groups is not significant. Red indicates the corresponding microbiota is significantly abundant in the CJD group. Green indicates the corresponding microbiota is significantly abundant in the control group. **(B)** We set LDA score > 3 as a threshold and gut microbiota with LDA score above the threshold was shown in this figure. p, phylum; c, class; o, order; f, family; g, genus.

We further confirmed the data above *via* LEfSe analysis (Figures 3A,B). A significant increase was observed in the Actinobacteria and Fusobacteria taxa according to the cladogram. The LDA score of the microbiota was also evaluated, and those with LDA score > 3 are shown in Figure 3B.

### Function Analysis of Gut Microbiota in CJD Group and Control Group

To investigate the functional alteration of gut microbiota between the CJD group and the control group, KEGG analysis *via* PICRUSt2 (v2.3.0) was performed. Five KEGG pathways were significantly altered in the CJD group, compared to the control group using the level II KEGG pathway, as shown in Table 3. When tracing these KEGG level II pathways to their corresponding KEGG level I pathway, it showed that the gut microbiota in the CJD group presented a potential linkage in human disease, metabolism, environmental information processing, and organismal systems. Compared to the control group, the enriched KEGG level II pathway in the CJD group is associated with immune disease, neurodegenerative disease, signaling molecules and interaction, xenobiotic biodegradation, and metabolism. The immune system decreased in the CJD group when observing the KEGG level II pathway. We also analyzed the KEGG level III pathway in both groups (Supplementary Table). The CJD group showed enrichment in the majority of xenobiotic biodegradation and metabolism, metabolism of terpenoids and polyketides, lipid metabolism, energy metabolism, immune disease, neurodegenerative disease, replication and repair, and signaling molecules and interaction, while decreases in carbohydrate metabolism, metabolism of cofactors and vitamins, translation and folding, and sorting and degradation.

**Table 3:** KEGG II pathway that were significantly altered in the CJD patients.

**KEGG function level I**	**KEGG function level II**	**CJD patients**	**Healthy controls**	***p***
Human Diseases	Immune disease	0.0000145	0	<0.001
Human Diseases	Neurodegenerative disease	0.0021246	0.0000779	0.001
Metabolism	Xenobiotics biodegradation and metabolism	3.3008108	1.1663813	0.002
Environmental Information Processing	Signaling molecules and interaction	0.0000083	0	0.013
Organismal Systems	Immune system	0.0719617	0.0814560	0.034

### Association of Gut Microbiota With Clinical Profile

**Figure 4:**
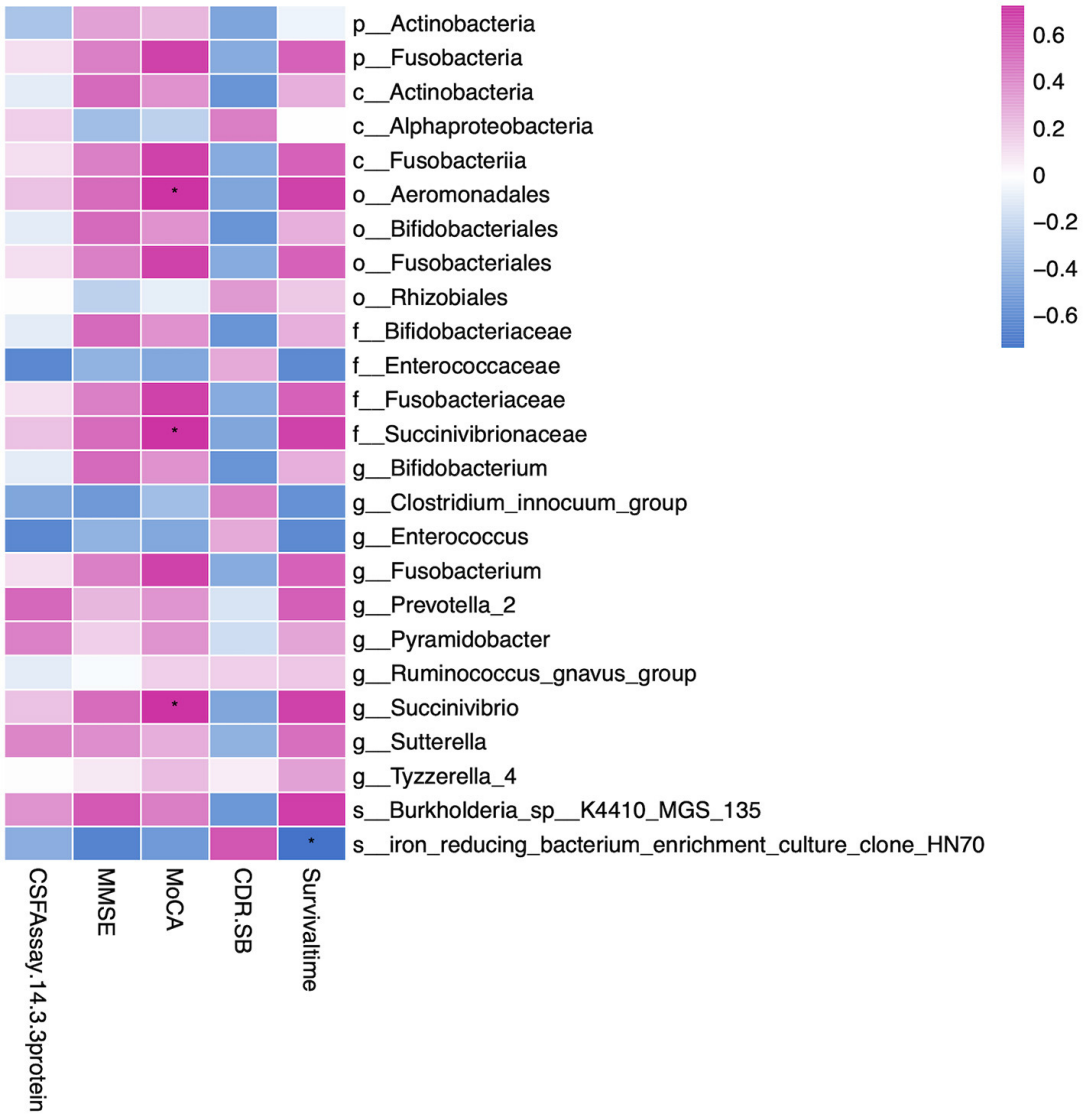
Heatmap showing the correlation of gut microbiota and clinical profiles, namely, MMSE, MoCA, CDR-SB, CSF 14-3-3 protein, and survival time. We found order Aeromonadales, family Succinivibrionaceae, and genus Succinivibrio were positively correlated to the MoCA rating scale. In addition, iron-reducing bacterium (clone HN70) was negatively related to the patient survival time. The correlation was calculated by the Spearman algorithm, and the heatmap was drawn by the ggplot2 package in R software. Red represents a positive correlation, and blue represents a negative correlation. ^*^Indicates the correlation is significant (*p* < 0.05). MMSE, Mini-Mental State Examination; MoCA, Montreal Cognitive Assessment; CDR-SB, Clinical Dementia Rating scale Sum of Boxes; p, phylum; c, class; o, order; f, family; g, genus; s, species. * represent p value < 0.05.

To explore the potential relationship between gut microbiota and clinical characteristics, we performed the correlation analysis among the microbiota of significance and CJD clinical profile. As shown in Figure 4, order Aeromonadales (*r*: 0.72, *p* value = 0.02) and its family Succinivibrionaceae (*r*: 0.72, *p* value = 0.02) and genus Succinivibrio (*r*: 0.72, *p* value = 0.02) were positively related to MoCA. Other scaling systems, namely, CDR, SB, and MMSE, were also influenced by the microbiota, but the association did not reach the level of significance. We also investigate the association between certain microbiota and patients with CJD survival time. Accordingly, we identified that the iron-reducing bacterium (clone HN70) was negatively related to the patient survival time (*r*: −0.74, *p* value = 0.03). Furthermore, we analyzed the association of gut microbiota with CSF 14-3-3 protein, which serves as a standard test for CJD diagnosis (28). However, no significant association between CSF 14-3-3 protein and gut microbiota was identified.

## Discussion

Although gut microbiota alteration has been acknowledged as an important factor in neurodegenerative diseases such as AD and PD, gut microbiota alteration in prion disease was seldom reported. Several studies about the gut microbiota in a prion mice model showed that the gut microbiota was imbalanced (22). However, gut microbiota alterations in humans with prion disease have not been reported thus far.

In this study, we performed 16S rRNA sequencing of fecal samples harvested from 10 patients with CJD and 10 matched healthy individuals to compare the gut microbiota composition between these two groups. Structurally, compared to the control group, we found that a higher gut microbiota abundance was identified in the CJD group. We also found that the gut microbiota in the CJD group showed a marked variation at a different biological taxonomic level. Phyla Actinobacteria and Fusobacteria were the 2 main phyla that were altered in the CJD group. More importantly, the presence of certain microbiota was associated with the clinical performance and survival time of patients with CJD.

According to the PLS-DA analysis results, patients with CJD and healthy controls were separated with no overlap, indicating the 2 groups could be distinctly differentiated. It showed a significant increase in the richness of the microbiota in the CJD group. A slight increase in the diversity of the microbiota was also identified in the CJD group, but it did not reach the level of significance. In a prion animal model, both the Chao1 and Shannon index decreased in the prion-infected mice, indicating reduced richness and diversity (22). This inconsistency may be explained by the fundamental difference between humans and mice. In addition, diet differences between mice and humans may also explain this inconsistency (29). Interestingly, there is some consistency and some contradictory findings when comparing our results to other studies in patients with PD and AD. A meta-analysis demonstrated that patients with PD have an overall increase in the Chao1 index, indicating a higher richness of the gut microbiota (30). However, in patients with AD, both the richness and the diversity of the gut microbiota are decreased (20, 21). It has also been suggested that the increased richness may be explained by the decrease of the commonly dominant microbes and the increase of the rare ones (30). Nevertheless, the definite change in the richness and diversity of gut microbiota remains unknown in prion diseases, and more studies are required.

Although we did not observe the decrease of phyla Firmicutes, the abundance of its class Negativicutes, and genus Roseburia, Holdemanella, and Lachnospiraceae_ND3007 group were decreased in our patients with CJD. In AD, a progressively decreased abundance was found in phyla Firmicutes from the healthy control group to the mild cognitive impairment group and the AD (20, 21). In PD, a decreased abundance of phyla Firmicutes is also observed when compared to the control group (18). One of the potential relationships between the decreased abundance of phyla Firmicutes and neurodegenerative disorder is that phyla Firmicutes are involved in short-chain fatty acid (SCFA) production (18). SCFA is beneficial to neurons, especially the brain microglial cells (16). In addition, SCFA also plays an important role in the permeability of the gut barrier (31). The decreased abundance of phyla Firmicutes in our patients with CJD may cause a shortage in SCFA, thereby leading to the dysregulation of both brain microglia cells and gut permeability. However, it is still unclear how gut permeability and microglial cell dysfunction may affect patients with CJD, and more studies are required in this field.

In the CJD group, 2 main microbe phyla, Actinobacteria and Fusobacteria, were increased significantly when compared to the control group. In the phylum Actinobacteria, its class Actinobacteria and one of its family Bifidobacteriaceae were increased in patients with CJD. While in phylum Fusobacteria, the increase in its class Fusobacteriia, family Fusobacteriaceae, and genus Fusobacterium were found in our results.

Phyla Actinobacteria are anaerobic gram-positive bacteria with branching rod shape, and it constitutes a minority (<10%) of the human gut microbiota (32, 33). The increase of phylum Actinobacteria is also identified in inflammatory bowel disease (34) and neuropathy, namely, AD (35) and autism (36). It is interesting that we found a significant abundance of phyla Actinobacteria, and its family Bifidobacteriaceae, in the CJD group, given that they are acknowledged as probiotics (33). Bifidobacteriaceae has been shown to produce an SCFA, which is beneficial to both the gut membrane and the nervous system (16, 37, 38). However, the significant abundance of it may still have harmful effects. It has been suggested that SCFA influences the gut barrier permeability in a concentration-dependent manner. A low concentration of butyrate decreases the gut permeability, while a high concentration of butyrate induces gut epithelium apoptosis, exerting an opposite effect (39). Thus, the increase in family Bifidobacteriaceae may produce excess butyrate, revealing its harmful effects on the gut. In AD, scholars indicate that the impaired gut permeability may facilitate the transfer of gut amyloid to the brain, initiating AD (40). It is possible that the increment of phylum Actinobacteria and its family Bifidobacteriaceae in patients with CJD may increase the gut permeability and the absorption of prion, which exacerbates the disease. Nevertheless, this hypothesis is limited to foodborne prion diseases such as variant CJD or Kuru disease. The exact role of phylum Actinobacteria in prion disease is still unknown, and more studies are still needed.

The increase of phylum Fusobacteria was also found at a different taxonomic level in patients with CJD. In general, phylum Fusobacteria is a gram-negative, rod-shaped like anaerobic bacteria, and normally present in the oral cavity (41). Fusobacteria has been found to play a critical role in several diseases, and it is repeatedly reported to be associated with colon cancer (42). In AD, individuals with elevated chronic oral Fusobacteria infection are more susceptible to AD because of the increased systemic inflammation (43). The Fusobacteria-induced systemic inflammation may have a potential role in the pathology of CJD (44, 45). M cell, which resides in the Peyer's patches of the gut, is an important vehicle to transport prion proteins into the host tissues (46, 47). Under a systemic inflammatory state, M cell density is significantly increased and leads to an augment in prion transfer, thereby exacerbating the course of prion disease (48, 49). The inflammation evoked by phylum Fusobacteria can accelerate prion disease *via* the microglia cell, which is critical in maintaining CNS homeostasis (50). In prion disease, it is suggested that the transfer of microglia from a “resting” state to an “activated” or a proinflammatory state plays an important role in the pathology of prion disease (51). Nevertheless, more research is required to study the pathological role of the phylum Fusobacteria in prion disease.

Several studies have demonstrated that gut microbiota correlates with clinical scaling. Gut microbiota under phylum Firmicutes is found strongly correlated with clinical scaling such as Unified Parkinson's Disease Rating Scale (UPDRS), Scales for Outcomes in Parkinson's Disease (SCOPA), and Non-Motor Symptom Questionnaire (NMSQ) in PD (52). In AD, phylum Proteobacteria and its subsequent class Gammaproteobacteria, order Enterobacteriales, and family Enterobacteriales are inversely correlated with MoCA significantly (20). In our CJD group, Alphaproteobacteria, Succinivibrionaceae, and Succinivibrio, which belong to the Phylum Proteobacteria, were positively associated with clinical dementia rating scale MoCA. This indicates that different members within this phylum Proteobacteria may have a distinct correlation with the clinical dementia state. We also performed correlation analysis between gut microbiota and CSF 14-3-3 protein and other dementia rating scales, namely, MMSE and CDR-SB. However, we did not find any significant correlation.

In our study, the KEGG pathway analysis revealed a broad gut microbiota functional alteration in patients with CJD. Immune and neurodegenerative diseases, immune system, signaling molecules interaction, and xenobiotics biodegradation metabolism were the major pathways enriched in the CJD group. The enrichment in immune disease and immune system pathways suggested that the gut microbiota in patients with CJD has fundamental influences on the immunity of these patients. This confirms the hypothesis that the alteration in the gut microbiota community is possible to induce the inflammation process and activate critical immune cells such as microglia in patients with CJD, which may potentially exacerbate the disease. Previous studies in AD show that gut microbiota is enriched in the pathways related to membrane transport and signal transduction, indicating the enhancement in the communication of gut microbiota with the host brain (20). In our result, although we did not find enrichment in ortholog-related to membrane transport and signal transduction, the enrichment in signaling molecules interaction suggested that gut microbiota in CJD may also display an enhanced communication with the host brain.

The functional alteration in CJD gut microbiota may provide new insight into the disease pathology and may suggest new avenues in the treatment of CJD. Therapy that targets the dysbiosis of the gut microbiota may be a new therapeutic strategy in treating this neurodegenerative disorder. Microbiota transfer therapy is shown to improve autism symptoms in children, and the long-term effects of this therapy have been confirmed (53). Another drug, GV-971, which is a mixture of acidic linear oligosaccharides, is capable of reducing the Aβ plaque deposition and reversing the gut microbiota dysbiosis in AD mice (54). In human subjects, GV-971 has also been shown as a safe and well-tolerated therapeutic strategy, according to the phase II clinical trial (55). In patients with CJD, gut microbiota transfer or drugs that target gut microbiota alteration may prolong the survival and improve the management of CJD patients.

There are several limitations of our study. First, samples of our patients were relatively small, and our study lacked ethnic diversity. The patients and controls were all Chinese, and we only recruited 10 patients with CJD and 10 healthy controls. Part of the reason is that CJD is a rare neurodegenerative disease, as such, it is hard to recruit a large sample size of patients. Meanwhile, the small number of patients may also increase the possibility of false-positive results in statistics. Second, it is hard to select the same type of patients with CJD. In our patient with CJD cohort, we recruited both sCJD and gCJD. Future studies may want to recruit the same type of patients with CJD having larger sample sizes and diverse ethnic backgrounds to evaluate results. Shared patient data among multiple neurological centers may be the best path forward in the study of CJD. Furthermore, our study is unable to differentiate the effect of the microbiome on CJD and the effect of CJD on the microbiome. Whether CJD itself will reshape the gut microbiota or the specific gut microbiota characteristics that make the individual vulnerable to the development of CJD remains unknown. Future longitudinal cohort studies are still needed.

In conclusion, we demonstrated that the gut microbiota of patients with CJD was significantly altered. Our results suggested that the gut microbiota was significantly altered in patients with CJD, which is in agreement with other studies in neurodegenerative disorders. Compared to the healthy controls, a marked increase in the phyla Actinobacteria and Fusobacteria was identified in the CJD cohort. Phyla Proteobacteria is also considered as increased in patients with CJD. Phyla Firmicutes, on the other hand, showed a decrease in abundance in patients with CJD. It is of note that the dysbiosis of the gut microbiota was related both to the severity and the progression of the disease. In the end, this is the first study focused on the gut microbiota alteration in patients with CJD, and we believe our results may provide new insight into the understanding of CJD and facilitate the development of therapy to prolong the survival of patients with CJD.

## Data Availability Statement

The datasets presented in this study can be found in online repositories. The names of the repository/repositories and accession number(s) can be found below: NCBI BioProject, accession number PRJNA791534.

## Ethics Statement

The studies involving human participants were reviewed and approved by Ethics Committee of Beijing Tongren Hospital, Capital Medical University, Beijing, China (TRECKY2021-179). The patients/participants provided their written informed consent to participate in this study.

## Author Contributions

YX drafted the initial manuscript and reviewed the literature. XL, ZZ, and WS were in charge of the patients' follow-up and assessments. FY and HG recruited the patients. QS performed the analysis. JW, YG, AY, and XD revised the manuscript. All authors of this manuscript have actively participated in the data acquisition and they all commented and approved the final version of the manuscript.

## Funding

This work was supported by the Open Project of State Key Laboratory of Infectious Disease Prevention and Control (2020SKLID311), the National Natural Science Foundation of China grant (81870888, 81301032, and 81771313), and the Capital Medical Development Research Fund (2018-2Z-1076).

## Conflict of Interest

The authors declare that the research was conducted in the absence of any commercial or financial relationships that could be construed as a potential conflict of interest.

## Publisher's Note

All claims expressed in this article are solely those of the authors and do not necessarily represent those of their affiliated organizations, or those of the publisher, the editors and the reviewers. Any product that may be evaluated in this article, or claim that may be made by its manufacturer, is not guaranteed or endorsed by the publisher.

## Abbreviations

AD: Alzheimer's disease
PD: Parkinson's disease
CJD: Creutzfeldt-Jakob disease
MMSE: Mini-Mental State Examination
MoCA: Montreal Cognitive Assessment
CDR-SB: Clinical Dementia Rating scale Sum of Boxes
PrP^Sc^: Aggregated form of prion protein
Aß: Amyloid beta
α-syn: Alpha-synuclein
rRNA: Ribosomal RNA
CSF: Central spinal fluid
OTUs: Operational taxonomic units
PLS-DA: Partial least squares discrimination analysis
CNS: Central nervous system
DOXY: Doxycycline.
