# Supplementary material for: Creutzfeldt-Jakob Disease: Alterations of Gut Microbiota
Source: Front Neurol. 2022 Apr 15;13:832599. doi: 10.3389/fneur.2022.832599 (PMC9051076; doi:10.3389/fneur.2022.832599)
Supplement: Supplementary file 1 [file Data_Sheet_1.DOCX]

**Supplementary figure**

A

B

Observed Species and PD whole tree between 2 groups

A. CJD patients showed a significantly increased Observed Species compared to the Healthy controls

B. CJD patients showed a significantly increased PD whole tree index compared to the Healthy controls

**Supplementary Table** KEGG level III pathway that were significantly altered in CJD patients

| kegg level I | kegg level II | kegg level III | CJD patients | Healthy controls | P value |
| --- | --- | --- | --- | --- | --- |
| Metabolism | Xenobiotics biodegradation and metabolism | Bisphenol degradation | 0.27339572 | 0 | <0.001 |
| Metabolism | Xenobiotics biodegradation and metabolism | Xylene degradation | 0.12061923 | 0 | <0.001 |
| Metabolism | Xenobiotics biodegradation and metabolism | Drug metabolism - other enzymes | 1.12830141 | 0 | <0.001 |
| Metabolism | Xenobiotics biodegradation and metabolism | Chloroalkane and chloroalkene degradation | 0.39077234 | 0.04366607 | 0.001 |
| Metabolism | Xenobiotics biodegradation and metabolism | Fluorobenzoate degradation | 0.02342001 | 0.03643528 | 0.004 |
| Metabolism | Xenobiotics biodegradation and metabolism | Toluene degradation | 0.23471291 | 0.055887 | 0.004 |
| Metabolism | Xenobiotics biodegradation and metabolism | Styrene degradation | 0.06419428 | 0.01460618 | 0.004 |
| Metabolism | Xenobiotics biodegradation and metabolism | Metabolism of xenobiotics by cytochrome P450 | 0.11280804 | 0.03608383 | 0.004 |
| Metabolism | Xenobiotics biodegradation and metabolism | Polycyclic aromatic hydrocarbon degradation | 0.00485142 | 0.00630215 | 0.015 |
| Metabolism | Lipid metabolism | Linoleic acid metabolism | 0.25851883 | 0 | <0.001 |
| Metabolism | Lipid metabolism | Arachidonic acid metabolism | 0.05380462 | 0 | <0.001 |
| Metabolism | Lipid metabolism | Steroid biosynthesis | 0.00388099 | 0.00010536 | 0.002 |
| Metabolism | Lipid metabolism | Fatty acid degradation | 0.39594494 | 0.32982987 | 0.010 |
| Metabolism | Metabolism of terpenoids and polyketides | Sesquiterpenoid and triterpenoid biosynthesis | 0.0000732 | 0 | <0.001 |
| Metabolism | Metabolism of terpenoids and polyketides | Biosynthesis of type II polyketide backbone | 0.00029435 | 0 | <0.001 |
| Metabolism | Metabolism of terpenoids and polyketides | Limonene and pinene degradation | 0.10709346 | 0 | <0.001 |
| Metabolism | Metabolism of terpenoids and polyketides | Carotenoid biosynthesis | 0.00414426 | 0.00042299 | 0.001 |
| Metabolism | Metabolism of terpenoids and polyketides | Tetracycline biosynthesis | 0.33952001 | 0 | 0.005 |
| Metabolism | Metabolism of terpenoids and polyketides | Biosynthesis of type II polyketide products | 0.00015811 | 0 | 0.005 |
| Metabolism | Metabolism of terpenoids and polyketides | Biosynthesis of 12-, 14- and 16-membered macrolides | 0.00000302 | 0 | 0.013 |
| Metabolism | Biosynthesis of other secondary metabolites | Betalain biosynthesis | 0.00248367 | 0 | <0.001 |
| Metabolism | Biosynthesis of other secondary metabolites | Flavonoid biosynthesis | 0.00510141 | 0.00039487 | 0.003 |
| Metabolism | Biosynthesis of other secondary metabolites | Novobiocin biosynthesis | 0.15926865 | 0 | 0.013 |
| Metabolism | Biosynthesis of other secondary metabolites | Isoflavonoid biosynthesis | 0.000026 | 0 | 0.013 |
| Metabolism | Amino acid metabolism | Arginine and proline metabolism | 0.72772558 | 0.7957675 | <0.001 |
| Metabolism | Amino acid metabolism | Tryptophan metabolism | 0.16040844 | 0.12715744 | 0.016 |
| Metabolism | Amino acid metabolism | Histidine metabolism | 1.26431459 | 1.37632971 | 0.019 |
| Metabolism | Carbohydrate metabolism | Glyoxylate and dicarboxylate metabolism | 0.66915821 | 0.75580749 | 0.001 |
| Metabolism | Carbohydrate metabolism | Galactose metabolism | 1.29692452 | 1.41811139 | 0.041 |
| Metabolism | Energy metabolism | Photosynthesis - antenna proteins | 0.00087953 | 0.0000066 | 0.001 |
| Metabolism | Energy metabolism | Photosynthesis | 0.51933857 | 0.07278557 | 0.002 |
| Metabolism | Energy metabolism | Oxidative phosphorylation | 0.42826634 | 0.46739338 | 0.008 |
| Metabolism | Metabolism of cofactors and vitamins | One carbon pool by folate | 1.67229009 | 1.78515612 | 0.016 |
| Metabolism | Metabolism of cofactors and vitamins | Riboflavin metabolism | 0.76655014 | 0.91568795 | 0.016 |
| Metabolism | Metabolism of cofactors and vitamins | Porphyrin and chlorophyll metabolism | 0.6158291 | 0.73084598 | 0.019 |
| Metabolism | Metabolism of cofactors and vitamins | Biotin metabolism | 1.33152886 | 1.6727374 | 0.028 |
| Cellular Processes | Cell growth and death | Meiosis - yeast | 0.00104212 | 0 | <0.001 |
| Cellular Processes | Transport and catabolism | Lysosome | 0.05676639 | 0 | 0.031 |
| Human Diseases | Immune disease | Systemic lupus erythematosus | 0.0000145 | 0 | <0.001 |
| Human Diseases | Neurodegenerative disease | Parkinson disease | 0.00212456 | 0.0000779 | 0.001 |
| Human Diseases | Infectious disease: bacterial | Epithelial cell signaling in Helicobacter pylori infection | 0.13296584 | 0.16054949 | 0.004 |
| Genetic Information Processing | Folding, sorting and degradation | Proteasome | 0.01497308 | 0.00203873 | 0.023 |
| Genetic Information Processing | Folding, sorting and degradation | Protein export | 1.34553353 | 1.41252868 | 0.041 |
| Genetic Information Processing | Translation | RNA transport | 0.05038054 | 0.06025757 | 0.010 |
| Genetic Information Processing | Replication and repair | Non-homologous end-joining | 0.02420575 | 0.00428734 | 0.028 |
| Environmental Information Processing | Signaling molecules and interaction | ECM-receptor interaction | 0.00000832 | 0 | 0.013 |
| Environmental Information Processing | Signal transduction | Plant hormone signal transduction | 0.00032274 | 0.0000313 | 0.002 |
| Organismal Systems | Immune system | NOD-like receptor signaling pathway | 0.07196174 | 0.08145598 | 0.034 |
